# Role of Polypropylene Fibres in Concrete Spalling Risk Mitigation in Fire and Test Methods of Fibres Effectiveness Evaluation

**DOI:** 10.3390/ma12233869

**Published:** 2019-11-23

**Authors:** Izabela Hager, Katarzyna Mróz

**Affiliations:** Faculty of Civil Engineering, Cracow University of Technology, 24 Warszawska St., 31-155 Cracow, Poland; kmroz@pk.edu.pl

**Keywords:** concrete spalling, temperature, fire, fibres, gas pore pressure

## Abstract

The explosive behaviour of concrete in fire is observed in rapidly heated concrete. The main factors controlling the occurrence of spalling are related to the material’s low porosity and high density as well as the limited ability to transport gases and liquids. Thus, for high-strength, ultrahigh-strength, and reactive powder concrete, the risk of spalling is much higher than for normal-strength concrete. The paper presents the discussion on the leading hypothesis concerning the occurrence of concrete spalling. Moreover, the methods for spalling prevention, such as polypropylene fibre application, which has been found to be an effective technological solution for preventing the occurrence of spalling, are presented. Various tests and testing protocols are used to screen concrete mixes propensity toward spalling and to evaluate the polypropylene fibres’ effectiveness in spalling risk mitigation. The most effective testing methods were selected and their advantages were presented in the paper. The review was based mainly on the authors’ experiences regarding high performance concrete, reactive powder concrete testing, and observations on the effect of polypropylene fibres on material behaviour at high temperature.

## 1. Introduction

During a fire, it is possible that concrete, which is an incombustible material [1], will behave explosively due to the occurrence of spalling. Spalling is defined as an unexpected loss of concrete cover that has a violent or non-violent character [2,3]. Spalling behaviour may endanger the load-bearing capacity of concrete elements because of a progressive cross-section loss and expose the steel reinforcement, which is sensitive to rising temperatures [4]. In a fire, this may lead to a decrease of the element capacity and even to its collapse [5]. Due to the fact that the effects of fire in concrete structures may be very severe, a number of experimental studies were carried out to find methods to reduce concrete propensity to spalling due to fire exposure [6,7,8,9]. The addition of polypropylene fibres (PP) to the concrete mix was found to give promising results. The polypropylene fibres being present in concrete does not significantly influence its mechanical and physical properties at a normal temperature [10]. However, when the temperature rises due to fire, the permeability of concrete with fibres increases as much as 50 times at 200 °C when compared to the concrete without fibres [11]. When fibres melt, a network of pores is created, which increases the concrete permeability. This facilitates the transport of water vapour, releases the water vapour pressure, and mitigates the concrete spalling risk in fire.

## 2. Concrete Spalling Due to Fire 

Spalling may take the form of a very slow detachment of the surface layer of the material, as well as of pieces of material, which break away in a rapid, explosive manner from the element [3]. Different mechanisms of spalling may lead to various types of spalling. The main differences between spalling forms are related to the time and frequency of the occurrence of spalling, the size of concrete pieces that are detached from the structure, and the energy that accompanies the spalling events in the event of a fire.

**Figure 1:**
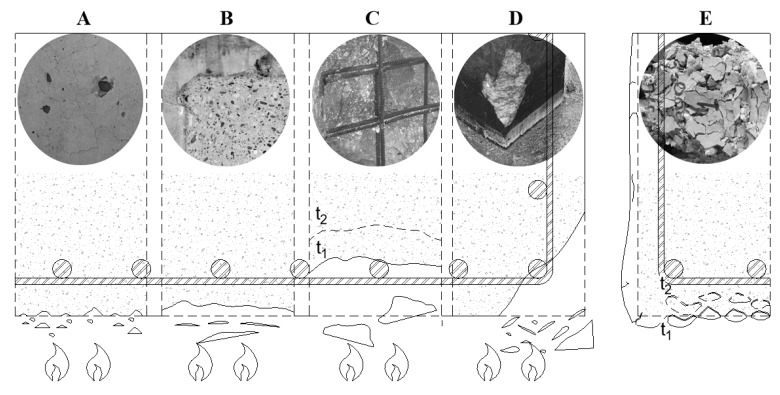
Forms of damage observed in various types of concrete spalling in fire conditions: (**A**) Aggregate spalling. (**B**) Surface spalling (progressive and continuous in nature). (**C**) Explosive spalling (progressive, discrete nature: t_1_, t_2_). (**D**) Corner spalling. (**E**) Surface peeling away during cooling, where t_1_ < t_2_.

Observations of fire damage to real structures and laboratory fire tests have made it possible to distinguish five different types of spalling, which were recently summarised by the authors [12]. The following subdivision was proposed, as shown in Figure 1. The times t_1_ and t_2_ indicated in the figure relate to different points in time showing the progressive character spalling type. In this subdivision, the corner (A), the aggregate (B), the surface (C), and the explosive spalling (D) were represented.

The occurrence of different types of spalling is accompanied by characteristic sounds—popping for the aggregate and surface spalling (A, B) and a loud bang for the explosive one (C). Some regularity in the occurrence of spalling, according to the type of spalling, was observed. During the first minutes of a fire or fire test, spalling is most likely to take place in the aggregate and surface. Then, after a few minutes, a loud bang of explosive behaviour appears, and afterwards, corner spalling takes place. Usually, aggregate spalling (A) does not affect the load-bearing capacity of a structure and does not lead to a significant cross-section loss. It is believed that the mechanism behind aggregate spalling is similar to a flaming stone procedure, which results from quartz transformation. Surface spalling (B) affects the thin and flat fragments of the near-surface layers of concrete elements, such as walls and ceilings, which become detached from the surface as a result of the tensile stress σ_T_ between the heated layer, which expands, and colder concrete layers. This is usually not dangerous for the load-bearing capacity of structures, since it generally does not result in the loss of a significant part of the cross-section and does not become exposed in reinforcing steel. Explosive spalling (C), which is accompanied by characteristic explosion sounds, constitutes a violent chipping off of concrete pieces, with large surface areas and spalling depth reaching up to around a dozen centimetres for a single piece of spalled concrete. Explosive spalling of concrete results in reinforcing steel becoming exposed and often results in the complete destruction of the concrete layer. Corner spalling (D) is observed in elements in which two orthogonal surfaces, which form a corner (in columns, beams, beam-column joints, beam-wall joints, etc.), are heated. In a typical fire, corner spalling usually takes place for about 30 min after the fire has started. Corners are heated more quickly, which results in higher thermal gradients at such points, and, consequently, corners are damaged more quickly as well. The peeling of concrete after a fire (E) characterises heated concrete, where dolomite and calcareous aggregates occur during the cooling of the structural element. This may take place a few hours or even days after the fire has ended. The peeling of damaged concrete layers after the fire is the result of an inversed temperature gradient during cooling. Nevertheless, the main cause of post-fire concrete peeling (concrete sloughing off) is the hydration of calcium oxide, formed during decomposition of portlandite, calcareous, and limestone aggregate, which is accompanied by the expansion of the reaction product [13].

### 2.1. Spalling Mechanism Hypothesis 

**Figure 2:**
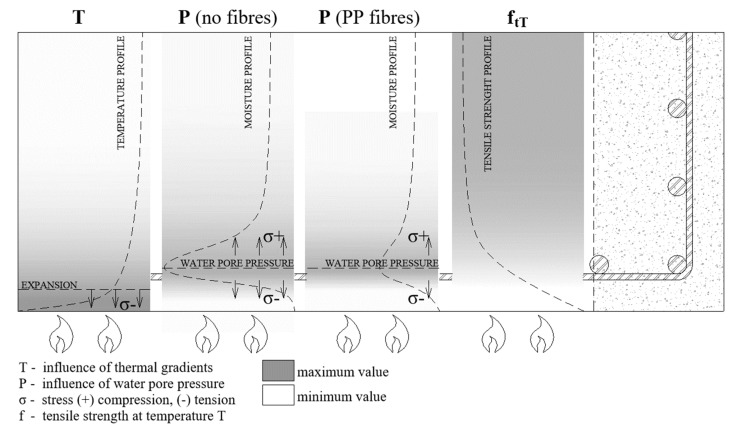
Mechanism of concrete spalling in fire and the pore pressure distribution in non-fibrous concrete and concrete with PP fibres.

Several hypotheses concerning the occurrence of spalling have been proposed [3,4]. Currently, it is believed that two simultaneously occurring and interrelated mechanisms are responsible for its occurrence, which are the thermal-mechanical and hygro-thermal mechanisms. The main reasons are believed to be linked to the thermal stresses (thermo-mechanical), originating from the thermal gradient in the element surface and cold inner layer, and to a water vapour pore pressure increase [14]. In Figure 2, we have represented the temperature and moisture profiles as well as the tensile strength change profile. Figure 2 represents a fire situation and an element heated on one side.

**Figure 3:**
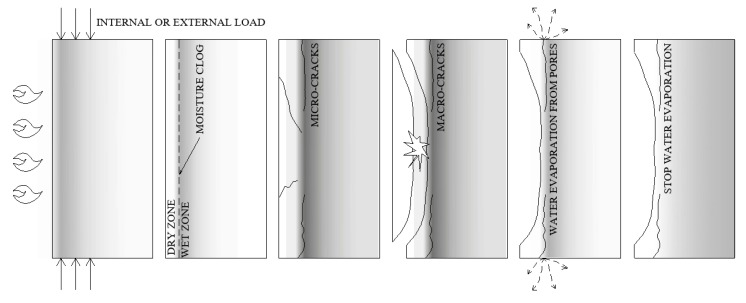
Development of the hydral processes in concrete exposed to fire.

During a fire, a temperature increase leads to concrete drying. The thermal gradient is also responsible for water migration. As the temperature increases, the water moves toward colder zones due to the thermo-capillary effect. Moreover, the water vapour that is produced during heating moves through an open network of concrete pores. The gas transport continues, until the thermodynamic conditions are satisfied for vapour condensation. Under this condition, a layer of material, saturated with water, is formed, which is called a moisture clog zone. The theory of a moisture clog was confirmed experimentally, in Reference [15], from an experiment, in which, after 15 min of heating, the concrete cubes were split, showing the concrete zones saturated with water. Moreover, the advantages of neutron tomography have also been proven in studying the mechanism of the phenomenon [16] and allowing for moisture movement, as well as the local moisture accumulation, in a heated concrete specimen observation. Under those conditions, the water vapour pore pressure starts to increase, and the pressure peak is located at the saturated layer. The pressure of the water vapour (P) is exerted on the material skeleton and reaches a maximum level in the water-clogged zone. When the tensile stress increases to a critical level, which is higher than the tensile strength of concrete, spalling may begin. In normal-strength concrete (NSC), the gas permeability measured by authors is 2.22E-16 [17]. Therefore, the water vapour moves with ease through the porous network and evaporates quickly. In denser and less permeable concrete, the moisture clog is generated sooner and closer to the heated surface. A fully saturated and almost impermeable layer of the material moves through the material, which we have represented in the pictogram shown in Figure 3. After some time, in the concrete that is most exposed to a temperature increase, a dry zone is formed, in which the cement paste is dehydrated. Subsequently, a drying zone, where no free water is present, adjacent to the saturated and impermeable concrete layer, is formed.

As a consequence, the water-clogged zone is more prone to forming in high-strength concretes (HSC) and ultra-high-performance concretes (UHPC), in which the permeability is 0.52E-17 [17]. Due to the low permeability of the material, a water clog zone will form quickly. Using the mechanism of spalling in an element heated on one face, we have represented it in Figure 2, which also takes into account the material strength reduction in temperature. This is approximately 120 °C, as reported in authors’ research [18]. The reduction of the mechanical performance of concrete is mainly due to thehydral gradient inside the heated concrete element or specimen. 

It needs to be highlighted that the external load has a critical effect on the spalling behaviour of a concrete element. It was observed that the external compressive load or pre-stress enhances the risk of spalling [19,20,21]. An external compressive load might be generated during the fire, as a consequence of the restrained element expansion. The restraint may be due to the presence of other elements of the structure or the cold part of the material surrounding the heated zone (localised fire). In the case of elements that are tense, it was noticed that crack development significantly reduces the occurrence of spalling events [6]. The crack formation allows the water vapour to escape and the gas pore pressure to be released. Concrete spalling is the result of pore pressure occurrence, compression in the zone exposed to fire, and internal cracks developing in the plane of the heated surface [3].

### 2.2. Pore Pressure Measurements 

**Figure 4:**
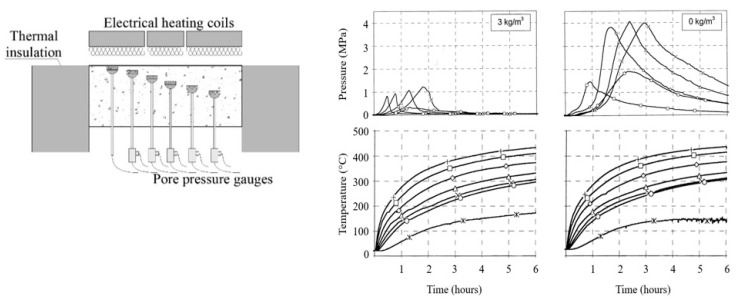
**Left**: PTM Kalifa set-up [10], **right**: Pressure and temperature measurements in heated HPC concrete: **Left**—with PP fibres, **right**—without PP fibres [10].

The gas pore pressures can be measured while heating using pressure gages embedded in the concrete element during casting. Kalifa et al. successfully measured the gas pore pressure and temperature development during heating [22], reporting that the water vapour pore pressure is much higher in HPC than in NSC. The values measured were 3.8 MPa and 1.8 MPa for HPC and NSC, respectively. PTM tests (PTM- Pressure, Temperature, Mass change) allow for the mass loss of heated slab (M), temperature (T), and the pore pressure (P) measurements. Specially designed sintered metal gauges are placed in a concrete element at different depths and allow pore pressure and temperature changes to be measured at the same time. The specimen, which is a small slab (0.3 × 0.3 × 0.12 m^3^), is placed horizontally and is directly exposed to an elevated temperature on the upper surface using radiant heaters, as shown in Figure 4 [10]. Further experiments have confirmed that, in concrete with a lower moisture content, the measured pressure is lower [23]. However, in a fully saturated specimen, this tendency is not found. The pressure is released quite quickly due to the macro cracking of concrete, which was observed during the experiment [23]. Recently, it was also reported, in Reference [24], that, when very rapid heating (120 °C/min) is applied, the developed pressures are three times smaller than those of concrete heated with a rate of heating at 10 °C/min. 

The instrumentation and measurements in PTM tests are very sophisticated. Today, PTM is considered to be the most advanced material testing method, helping the research community to observe the pore pressure development in fire conditions and to understand the physical mechanism behind it. This technique was also used to measure the effectiveness of PP fibre addition in pore pressure build-up in heated concrete and associate it with the spalling propensity of certain concretes. In Figure 4 [10], the PP fibre action is presented, which allows the gas pore pressure to be released effectively and reduces the pressure picks to 1 MPa, with 3 kg/m^3^ of fibrillated PP fibres. The PTM test also serves as a calibration test for all thermo-hydro-mechanical models that are able to predict the water clog presence and the gas pore pressure increase.

### 2.3. Standard Spalling Prevention Methods and Recommendations 

The construction element in case of fire must present a load-bearing capacity for a certain time to allow for the evacuation of the building. Moreover, the fire brigade must conduct the intervention in safe conditions. In some situations, the spalling behaviour of concrete may jeopardise the safety of concrete works. For beams, slabs, and all tensile members, crack occurrence may lead to a release of gas pore pressure, which decreases the spalling risk. The surfaces that are directly exposed to fire and work under compression require verification with regard to the spalling of concrete cover. In Reference [25], it is shown that, when the moisture content of concrete is lower than 3% by weight, explosive spalling is unlikely to occur. Nevertheless, at more than 3%, a more accurate assessment of the moisture content, nature of the aggregate, permeability of the concrete, and heating rate should be considered in order to check the spalling risk. The moisture content threshold may differ from 3%, and this information can be found in the National Annex to EC2. The recommended value is 3%.

For all structural elements made of HPC, and for concretes containing silica fume in an amount higher than 6% at the design stage, the risk of spalling should be taken into account. One of the following four methods should be applied. In the first prevention method, a reinforcement mesh, with a nominal cover of 15 mm consisting of wires of a diameter of 2 mm and a pitch size lower than 50 × 50 mm^2^, is used. Additionally, the nominal cover of the main reinforcement should be 40 mm. In the next method, a concrete mix should be used, to which it has been demonstrated experimentally that no spalling occurs under fire exposure. The following technique consists of the application of protective layers, to which it is demonstrated that no spalling of concrete occurs under fire exposure.

Other spalling prevention techniques have been tested on existing structures, such as the application of heat protection panels, made of wool or gypsum, that slow down the heating process, which is a commonly used solution [26]. This type of protection is considered to be passive fire protection. In the case of new concrete structures, steel fibre addition was proposed to increase the tensile strength of concrete and steel mesh protection in order to reinforce the outer layer of the concrete structural element, but the effectiveness of this method is limited. In order to prevent concrete spalling in fire, the application of organic fibres is proven to be the best solution. For this purpose, polypropylene (PP) fibres are added to the concrete mix. The fibres are used in an amount of 0.1% to 0.2% of the volume of the concrete. Both types—fibrillated and monofilament fibres—were reported to be efficient in spalling mitigation. The fibrillated fibres are obtained by cutting polypropylene sheets. Therefore, their cross-section is rectangular. Of the presented fibres, with a section of 50 × 100 μm, various quantities were investigated, from 0.5 to 3.0 kg/m^3^ of concrete. Nevertheless, for a high PP fibre content, a mixing problem is observed, and homogeneity in the concrete mix is difficult to obtain. It was reported that an increase in the total volume of the cement paste content by about 15% enables the fibres to be covered with cement paste. The effectiveness of PP fibre addition, as a technological solution that prevents spalling, was confirmed by numerous experimental studies [27,28,29]. Moreover, the addition of 2 kg/m^3^ of fibres is also recommended by the EUROCODE 2 [4] standard to avoid fire spalling.

## 3. Polypropylene Fibre Action 

### 3.1. PP Fibre Behaviour at High Temperatures

PP fibres melt at a temperature of around 170 °C, i.e., lower than the temperature at which the maximum water vapour pressure in concrete is reached (190–260 °C). In the market, low-temperature melting PB Eurofiber HPR (Heat Prompt Reaction) fibres can be found, and, for this fibre variation, melting occurs more quickly because their melting temperature is lower.

**Figure 5:**
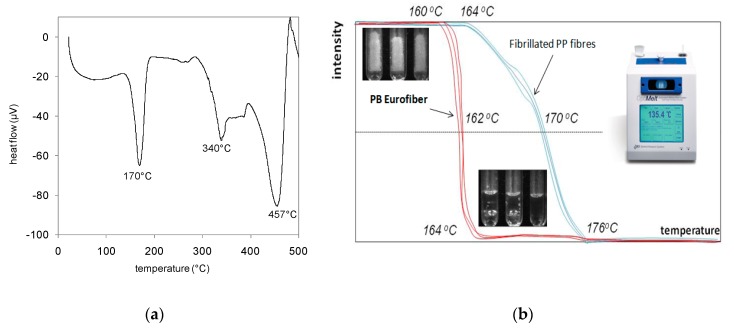
(**a**) Differential thermal analysis of polypropylene fibres and (**b**) observation of the melting process and the melting temperature range for polypropylene fibres.

At 350 °C, polypropylene burns and turns into CO_2_ and H_2_O. In Figure 5a, a differential thermal analysis of polypropylene fibres was presented [10]. The peaks on the curve correspond to the melting point (171 °C), evaporation temperature (341 °C), and carbonation temperature (456 °C). The fibres melt at 171 °C and are totally or partially absorbed by the porous network of the cement matrix. At 341 °C, the polypropylene vaporises. The melting temperature can be evaluated using an Automated Melting Point Apparatus MPA100, which allows the melting process to be observed and the range of temperatures at which melting takes place to be precisely determined, as shown in Figure 5b.

In our study, we compared two PP fibres types, known as PP fibrillated polypropylene fibres and PB Eurofiber, with a low melting temperature, which have shown advantageous melting temperatures (162 °C compared to 170 °C for fibrillated PP fibres).

**Figure 6:**
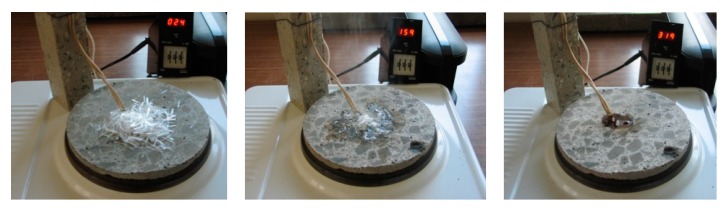
Fibrillated polypropylene fibres melting and the absorption of polypropylene by the cement matrix and PP burning.

After melting, the polypropylene is partly absorbed by the cement matrix. We have represented it in an experiment, in which several fibres are heated on a concrete disk. The thermocouple shows the temperature at the location of the fibres, as shown in Figure 6.

Similarly, when fibres melt and are absorbed by a cement matrix, a network of pores is created, which increases the concrete permeability. This facilitates the transport of water vapour. The channels that remain after fibres have melted add up to the natural porosity of the cement paste and the porosity of the interfacial transition zone (ITZ) between the cement paste and aggregates.

Nevertheless, as reported by Kalifa et al. [10], several micro-cracks around fibres have been observed. The cracks observed were 1 μm in size for concrete with fibres and 10 μm in size for non-fibrous concrete. Mercury intrusion porosity measurements confirmed a significant increase in the quantity of pores with diameters of less than 1 μm in HPC concrete, when fibres were heated to 180 °C. The change observed does not stem from the additional porosity caused by the melting of polypropylene fibres but is, instead, the result of an increase in the micro-cracking of the heated concrete with PP fibres.

**Figure 7:**
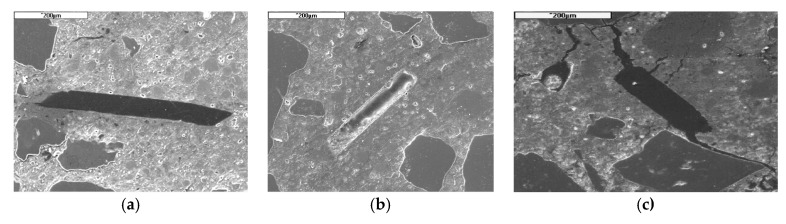
Polypropylene fibre cross-section in concrete (**a**) non-heated, (**b**) heated to 150 °C and (**c**) after 600 °C. SEM image at 50x.

Two hypotheses concerning this additional cracking in fibrous concrete were put forward. The first is that the fibres, while being heated, dilate by 7–10% [29], which can generate local stresses and nucleation of cracks. The second is that the fibre bed promotes the local formation of cracks and encourages cracking, which we have represented in Figure 7. The crack formation around the fibres has the beneficial effect of reducing the water vapour pressure in the concrete pores. It is believed that the fibres’ melted network adds up to the natural porosity of the material, i.e., as estimated by Bentz [30] from a numerical analysis. The amount of PP fibres when added to the concrete allow the percolation threshold to reach 0.1% of fibres, relative to the concrete volume.

### 3.2. Change of Transport Properties in Heated Concretes with PP Fibres

In order to better understand the thermo-hydric process leading to the spalling phenomenon and explain the role of polypropylene fibres in spalling mitigation, Kalifa’s PTM experiment was employed. In this small-scale test, the influence of PP fibres on temperature distribution, pore pressure build-up, and mass loss in a heated specimen may be tested. According to the experiments conducted on concrete (f_c_ = 100MPa), with different amounts of PP fibres (0–3kg/m^3^), the amount of 1.1 kg/m^3^ in concrete heated on one side leads to a pore pressure development of about 2 MPa, which is close to the one recorded on NSC (where spalling usually does not occur). Pressure and permeability measurements suggest that a dosage of around 1 kg/m^3^ should be sufficient for this type of concrete and within this range of temperatures. This value may not be sufficient for other types of concrete, with a smaller porosity, as in UHPC [10]. Similarly, Mindeguia et al., by performing a PTM test, showed that, in normal-strength concretes (f_c_ = 40 MPa and f_c_ = 60 MPa) exposed to moderate heating, the addition of 2 kg/m^3^ of PP fibres caused a three-fold reduction in the pore pressure [31].

Experimental investigations have demonstrated the effectiveness of fibres as a means of reducing the water vapour pressure in concrete. PP fibres at an amount of about 0.2% of the volume makes it possible to effectively reduce the internal pressures. The addition of 1.75 kg/m^3^ results in a 70% reduction in the value of the maximum pressure, which was observed when comparing it with a non-fibrous reference concrete. The incorporation of the fibres significantly reduces the values of the maximum pore pressures and decreases the temperature of the pressure peaks (180–200 °C). It has also been noted that the effect of fibres does not increase significantly when the fibre content is higher than 1.75 kg/m^3^.

There is an alternative explanation for the observed positive effect of PP fibres. Their introduction to concrete decreases the strength after melting by introducing a supplementary porosity that affects the strength. The reduced tensile strength may allow the formation of thermal cracks in the cross-section by unloading the thermal compression stresses necessary for spalling on the surface layer. This theory, highlighted in Reference [6], explains why fibres reduce the spalling of slabs and unloaded specimens instead of loaded columns, which are made of the same material. 

The permeability of concretes with fibres increases rapidly with temperature. In Reference [11], different lengths and quantities of fibres were used in order to choose the best PP fibre option in terms of size and quantity, which provides an essential permeability increase. In this study, PP fibres with a specific gravity of 0.91 g/cm^3^, three different amount of PP fibres (0, 0.9 and 1.8 kg/m^3^) and three lengths (6, 12 and 19 mm) were used. The five series of concrete were tested, designated as “HPC_fibredosage_fibre length.” The increase of the heated concrete gas permeability was measured using the Cembureau method, described in detail in Reference [17]. The study has shown the effect of the length and content of PP fibres on the relative permeability of heated HPCs.

**Figure 8:**
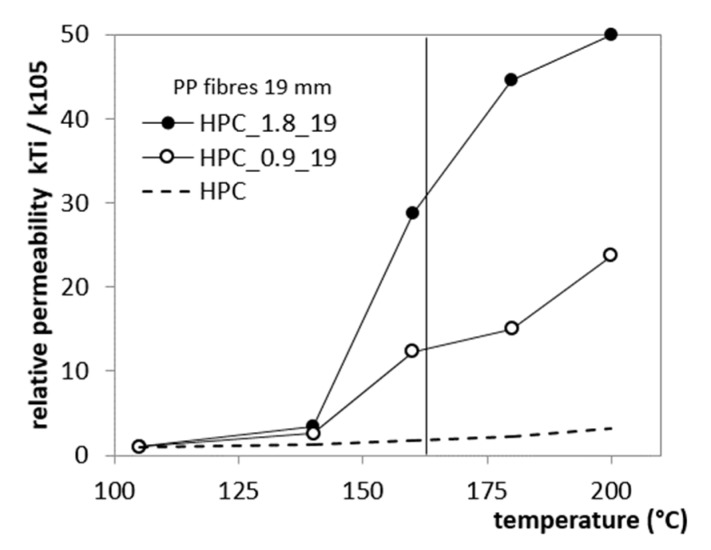
Relative permeability change of heated HPC and the effect of PP fibre amounts of 0, 0.9 and 1.8 kg/m^3^ [11].

**Figure 9:**
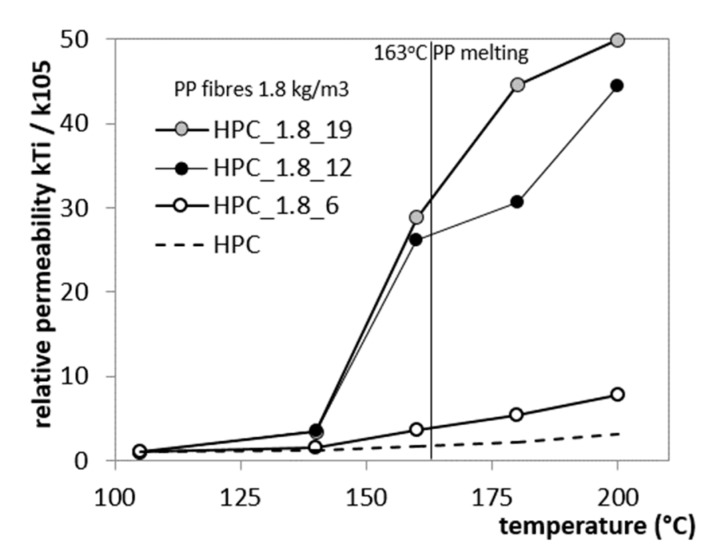
Effect of fibre length on the HPC relative permeability of heated concrete with 1.8 kg/m^3^ of PP fibres with lengths of 6, 12 and 19 mm [11].

The addition of polypropylene fibres in amounts of 0.9 and 1.8 kg/m^3^ does not strongly affect the initial properties of concrete (Figure 8). The sharp permeability increase was observed between two measuring points at 140 °C and 160 °C. A significant increase in residual permeability takes place after heating to 160 °C, which is a temperature of 3 degrees lower than the melting temperature of the fibres used. The sharp permeability increase after exposure to 160 °C may be caused by partial absorption of fibres by cement matrix and due to debonding of fibres from the cement matrix. The length of PP fibres hasa significant impact on permeability development, as shown in Figure 9. Short fibres (6 mm) seem to be inefficient in creating an interconnected and permeable pore network. Although the total dosage of PP fibres was equal, the short fibres of 6 mm provide four-fold lower permeability than fibres of 12 mm in length and eight-fold lower than 19-mm long fibres.

**Figure 10:**
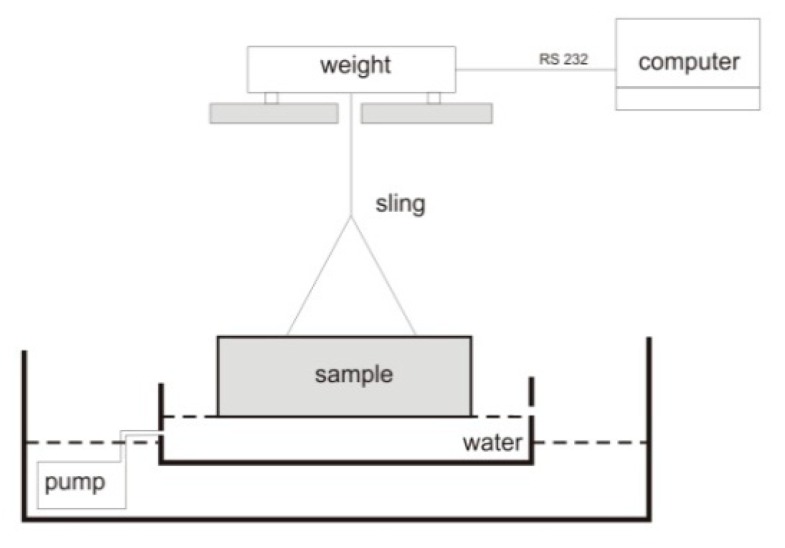
The water absorption measurement set-up.

**Figure 11:**
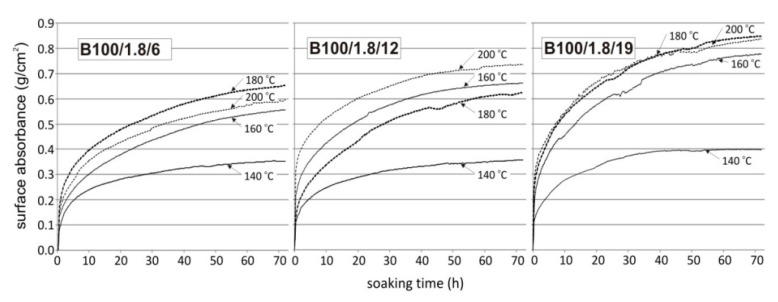
The HPC water absorption for concretes heated to 140, 160, 180 and 200 °C, with 1.8 kg/m^3^ of PP fibres. Fibre lengths: 6, 12 and 19 mm [32].

A practical method for additional open porosity evaluation in heated concretes with PP fibres is the surface water absorption test. Concrete discs are heated to different temperature levels, which is close to the melting temperature (163 °C in this research, or 160, 180, and 200 °C). The test consisted of measuring the mass gain of the specimen, which is in contact with water. During the test, the water level remained constant, and an increase in the sample mass was recorded. The measurement set-up is shown in Figure 10. In Figure 11, the results of the investigations on the influence of the PP fibre length on the transport properties of HPC were presented (designated as “HPC100_fibre dosage_fibre length”). The water surface absorption was tested, and it showed the increase of the open porosity volume and capillary porosity of the cement matrix. Consecutive measurements of the water absorption of heated concretes over time have enabled us to show that the porosity network was more developed in concretes in which the longer fibres, favouring percolation, were used (Figure 11).

### 3.3. The Influence of PP Fibres on the Mechanical Performance of Heated Concrete 

**Figure 12:**
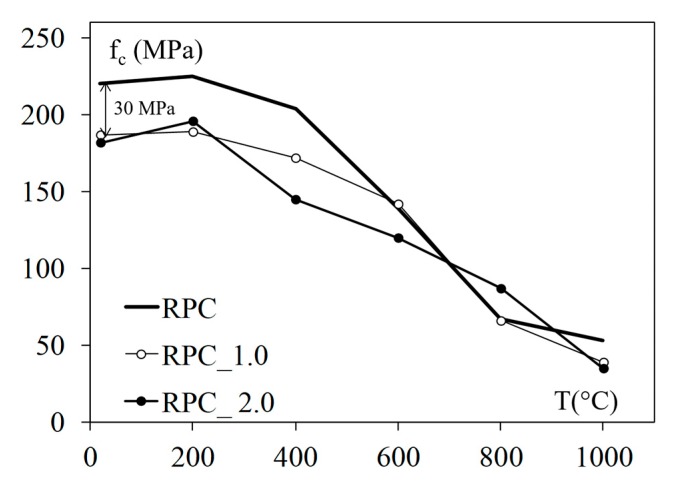
Theinfluence of heating temperature on changes in the compressive strength of RPC and RPCs with PP fibres [28].

According to some sources, PP fibres at amounts of 0.1% to 0.2% lead to a decrease in the initial strength of concrete by 10% to 15%. The strength decrease is due to the lower stiffness of the fibres in the cementitious matrix. Moreover, the material compaction of the higher fibres’ content is more complicated. Therefore, the concrete porosity may be the cause of the fibrous concrete mechanical performance decrease. The influence of the heating temperature on the changes in the compressive strength of the reactive powder concretes, with and without PP fibres, was studied [28], and the results of this investigation are presented in Figure 12. The RPC concretes with 1 and 2 kg/m^3^ of short PP fibres are presented, and their initial difference in strength was 15% (30 MPa).

**Figure 13:**
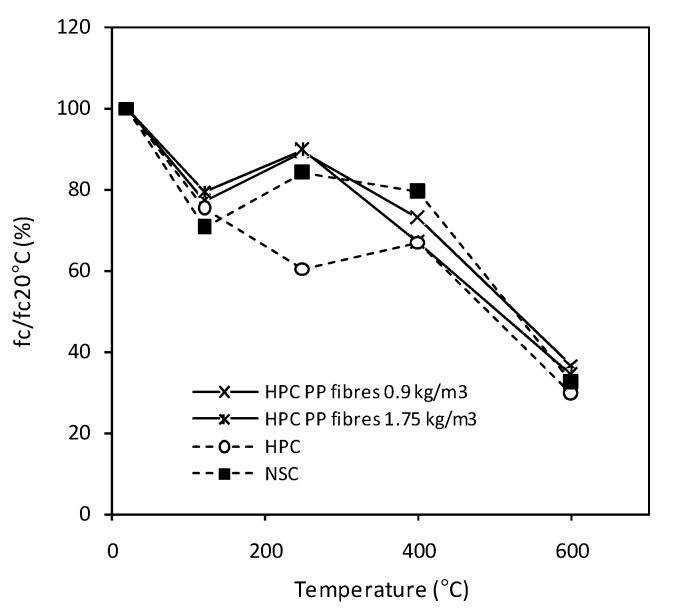
Relative values of compressive strength for normal-strength concrete and HPC, with 0, 0.9, and 1.75 kg/m^3^ of fibres [33].

In Figure 13, the relative values of the compressive strength evolution for tested heated HPC, with 0, 0.9 and 1.75 kg/m^3^ of fibres, are presented [33]. The applied heating rate was 1°C/min, and the tests were performed 2 h after a plateau had been reached. The relative values of the compressive strength evolution (relative values refer to the compressive strength of the non-heated material) for HPC with PP fibres were somewhat identical to those of ordinary concrete (f_c_ = 39.5 MPa), which confirms that the PP fibres contribute to the permeability increase.

The results for the compressive strength, tested at 120 °C, presents decreases of about 20%. This was explained by the presence of a hydral gradient in the middle of the specimen where the core of the specimen is saturated with water, and the surface has dried out. The observed decrease in the mechanical performance in the water clog zone is considered to contribute to the spalling behaviour. When the melting temperature of fibres is reached, water evaporation is possible. Therefore, for fibrous HPC and ordinary concrete, a partial strength restoration was observed at 250 °C. The concrete performance reaches 35% of the initial compressive strength, when the temperature reaches 600 °C for all tested materials.

## 4. Testing Methods for the Evaluation of the Effectiveness of PP Fibres in Spalling Mitigation

The assessment of concrete behaviour at a high temperature is carried out using three types of testing methods at different levels of analysis: small-scale, medium-scale, and full-scale analysis. Small-scale tests serve to investigate the material behaviour of concrete exposed to an elevated temperature, i.e., the thermal strains, gas pore pressure, temperature gradient, or mechanical properties. Full-scale fire tests are carried out on full-sized concrete elements in a real situation, in which the boundary conditions, external load, and conditioning correspond to the design. A full-scale test, as the most representative test, shows the qualitative and quantitative behaviour of structural concrete elements subjected to fire. The medium-scale test is carried out on concrete slabs exposed to fire, with a surface area of ca. 1m^2^. The concrete specimens in medium-scale tests may be reinforced and loaded or restrained concrete, depending on the laboratory procedure. Medium-scale tests are performed as a screening test to verify the behaviour of specific concrete mixtures in fire conditions and select the one that does not tend to spall. To investigate the influence of polypropylene fibres on concrete spalling behaviour in fire, the different testing methods were developed, and the most interesting method was described. To date, no standardised document concerning the testing of the spalling behaviour of concrete has been proposed to unify the material testing methods in order to allow for the screening of concrete mixes.

**Figure 14:**
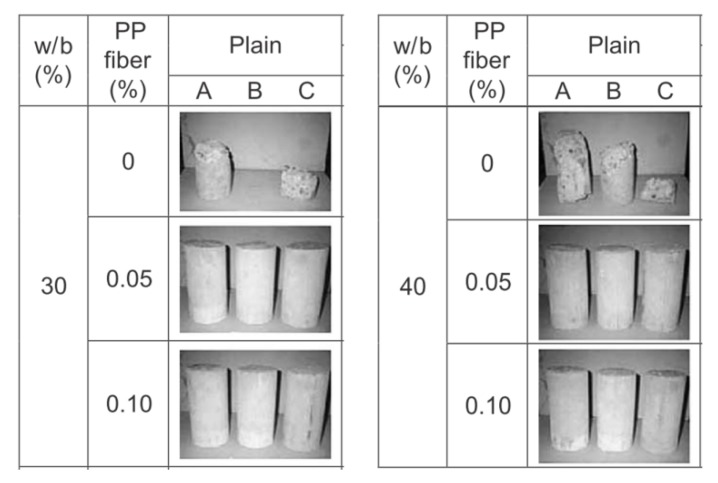
Results of simple small-scale tests on cylinders [9].

The assessment of the susceptibility of concrete to spalling can also be conducted using a simple small-scale test that relies on preparing laboratory samples (cylinders, prisms, or cubes), with and without PP fibres, and exposes them to an elevated temperature in a laboratory electric furnace. Such a test is commonly used to screen the concrete mix in order to check only if it spalls or if it is not under an elevated temperature. In those tests, samples are prepared with different types of fibres and dosages to optimise the minimum effective amount that reduces spalling and, at the same time, does not significantly affect fresh concrete properties and enable appropriate mixing and homogenization. A small, simple test using cylinders was carried out by Han [9]. Normal-strength concrete (f_c_ = 40MPa) with three amounts of polypropylene (PP) fibres—0.0, 0.45 and 0.90 kg/m^3^—was investigated for two types of concrete of different water to binder (w/b) ratios: 30% and 40%. The specimens were heated in a laboratory furnace with the heating curve KS F 2257 [9]. The results show that the addition of the smallest amount of PP fibres (0.45 kg/m^3^, 0.05% in volume) significantly reduces the spalling behaviour in tested concrete, as shown in Figure 14.

**Figure 15:**
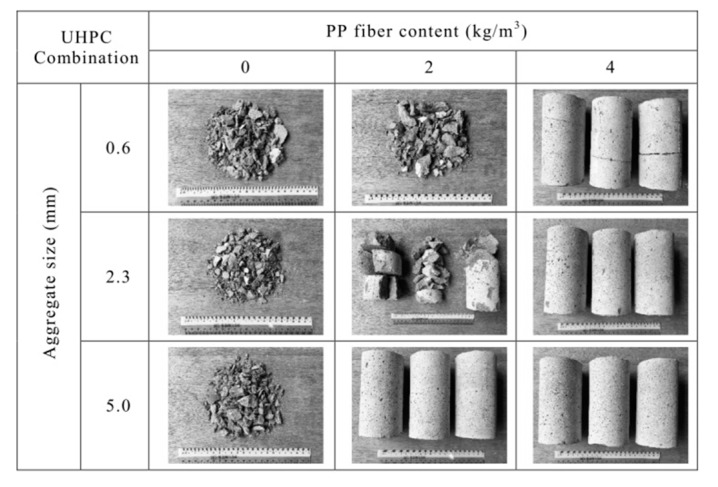
The results of a small, simple test in a laboratory furnace on concretes with different PP fibre dosages [34].

Similarly, small samples were also tested by Li [34] using Ultra-High-Performance Concretes (UHPCs, fc = 125MPa), with the addition of PP fibres at amounts of 0.0, 2.0 and 4.0 kg/m^3^. This research aimed to evaluate the aggregate size. A small, simple test on cylinders exposed to the ISO-834 [35] heating curve in a laboratory furnace showed that, in the case of UHPCs, the spalling elimination was possible only for 4 kg/m^3^ of PP fibres for each tested composition, as shown in Figure 15.

These tests are not considered to be representative of a fire situation involving construction projects when the material is under load. In order to develop a test that is more representative of a fire situation and to investigate the behaviour of the material from loaded concrete samples, heating the setups for specimens under compressive loads are used. Such a test can be helpful for assessing the changes in the mechanical properties of concrete caused by an elevated temperature, as well as to study the PP fibre dosage effect on the change of properties. 

**Figure 16:**
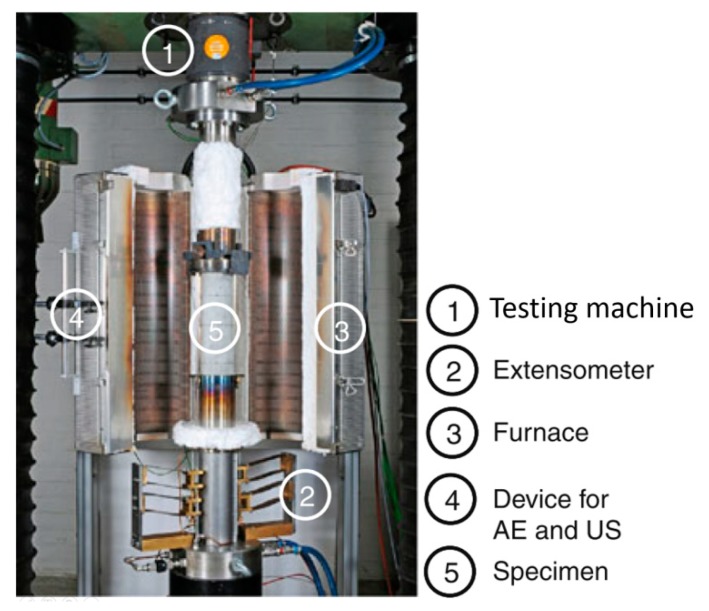
Example of a small sample test under a compressive load [36].

In several tests, an acoustic emission (AE) setup is additionally assembled near the specimen. Fire tests with an AE setup provide essential information on micro-cracking development that may differ for fibrous and non-fibrous concretes. Since micro-crack development is considered to enhance the moisture release and lower the pore pressure, information on it is essential for tests on the fire spalling of concrete. Huismann [36] tested cylinders made of HSC (f_c_ = 98 MPa) under an elevated temperature and uniaxial compressive load action, with 2 kg/m^3^ and without the addition of PP fibres. Cylindrical specimens of Ø100 mm and H300 mm were heated at 1°C/min and loaded with a constant ratio. The setup enabled the measurement of the temperature changes by thermocouples, axial deformations by an extensometer, and internal damages by AE records, as shown in Figure 16. Such small tests led to differences in the development of micro-cracks in the specimen to be investigated while heating and the changes in mechanical properties of the tested concrete to be shown. Huismann noticed that the addition of PP fibres resulted in more AE events between 200 and 250 °C and linked it with intensified micro-cracking and moisture release out of the specimen. Measurements of the transient strain enabled the micro-cracking development to be confirmed since the recorded strains were found to be higher for fibrous concrete than for non-fibrous concrete. Huismann also noticed that, in HSC with PP fibres heated to 600 °C, the cracks were thinner and more numerous than the HSC without PP fibres. Micro-cracks in fibrous concretes are considered to develop as a result of increasing the volume of melted PP fibres and the expansion of the fibre bed.

**Figure 17:**
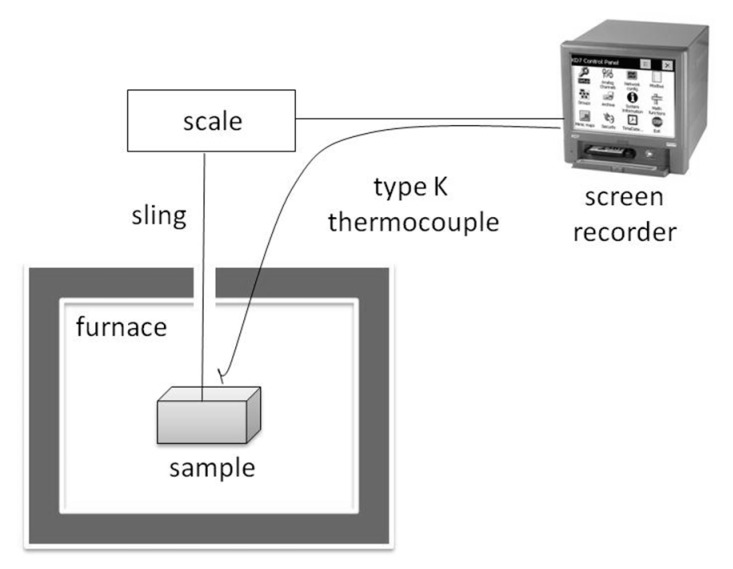
The mass loss set-up for spalling behaviour observation.

A simple weight loss technique was used to establish spalling events and sample mass loss during heating, as shown in the authors’ research [28]. The specimens were heated in an electric furnace. During the test, different heating ratios were applied to study the influence of this parameter on concrete spalling behaviour at a high temperature. The concrete samples were hung above the electric furnace, and the mass of the sample was continuously recorded during heating. The temperature was measured using a type K thermocouple placed in proximity to the sample’s surface. The measurement set-up is shown in Figure 17. From the experiment, the mass loss curve is traced, and the signal disruption corresponding to the sample explosion is recorded. Moreover, from the mass loss curve, the permeability of the material can be evaluated by analysing the kinetics of mass loss. For the concrete with PP fibres, when the polypropylene melts, the mass loss is more pronounced, and the mass loss rate is higher.

**Figure 18:**
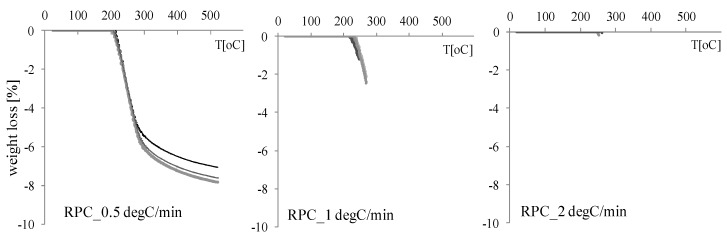
The mass loss results for the RPC concrete were heated with three heating rates: 0.5, 1.0, and 2.0 °C/min.

In Figure 18, the results for ultra-high-performance RPC reactive powder concrete were presented. During slow heating, i.e., with a heating rate of 0.5 °C/min, none of the four RPC specimens exploded. When the heating rate was increased to 1.0 °C/min in all four samples, spalling occurred at temperatures from 241 to 261 °C. As expected, a further increase of the heating rate (2 °C/min) also resulted in RPC showing spalling behaviour (T = 253–262 °C). As shown in Figure 18, an impulsive explosion occurs when the temperature near the surface of the specimen reaches 250 °C, practically without a mass change.

This experiment has clearly shown that, for RPC concretes, high-density cementitious materials with a compressive strength reached 250 MPa. In addition, the risk of spalling in fire is high and PP fibres may prevent spalling. For RPC with 1 kg/m^3^ of fibre, spalling risk occurs when the heating rate is 4 °C/min, and 2 kg/m^3^ of fibres prevent spalling, even when 8 °C/min is applied (Table 1).

The small material test does not provide information on element behaviour in fire. For cubes, cylinders, and prisms, heating is applied on all sides. In a real fire situation, heating on one side usually occurs. In order to more adequately reflect the stress state and boundary condition in a full-scale concrete element, medium-scale tests are commonly used on loaded or restrained concrete elements at a reduced scale. Cost-effectiveness is an essential aspect of screening concrete mixes for spalling propensity. Research laboratories commonly use the testing setup that employs gas burners to expose concrete to fire.

**Figure 19:**
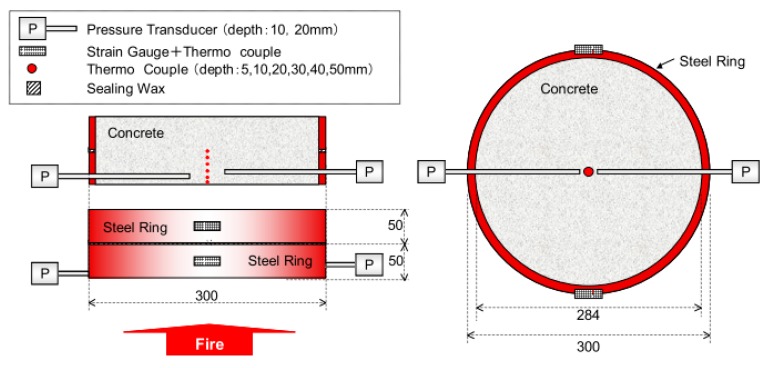
The scheme of the testing setup of the ring test for the fire spalling of concrete [37].

A useful example of a medium-scale test was proposed in Reference [37]. In this method, a circular concrete element is moulded in a steel ring, with an external diameter of 300 mm. The testing setup is developed in order to monitor the internal temperature, the gas pore pressure, and restrained stress of the concrete specimen. The element is placed on top of the gas furnace and heated from the bottom in a RABT 30 (RABT—Richtlinienfür die Ausstattung und den Betrieb von Straßentunneln) fire scenario, as shown in Figure 19. During the test, the steel ring confines the concrete that expands under an elevated temperature. While the concrete expands due to the elevated temperature, the thermal strains can be measured by recording the strains of the external steel rings. The thermal stresses of the concrete were recalculated from the measured strains of the steel ring at a particular temperature.

**Figure 20:**
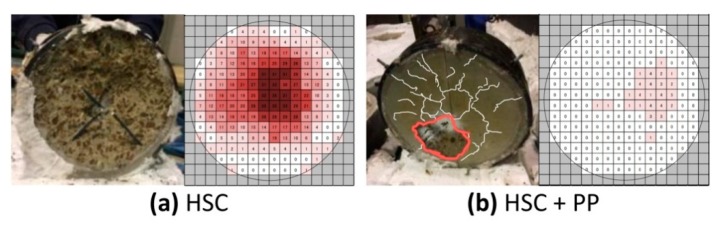
Results of the spalling tests for (**a**) plain HSC, and (**b**) HSC with PP fibres [38]. The advantages of the ring tests consist in easily casting and reproducing compressive loading in a similar way to the existing concrete structures, i.e., lateral compression. Such a test can be performed to screen the concrete mixture composition, before designing the concrete structures.

In 2018, the research team of Akasaka [38] presented comparative test results on the behaviour of HSC, with and without PP fibres, at 0.1% in terms of the volume. The specimens were heated in a RABT 30 fire scenario from the bottom. The results showed that the addition of 0.1% of PP fibres did not completely prevent spalling but reduced its extent from the maximum depth in non-fibrous HSC of 35 mm to 5 mm in the case of HSC with PP, as shown in Figure 20.

While considering the changes in the thermal stress of concrete, it was found that the stresses induced by HSC with PP are higher than those induced by non-fibrous HSC. This is due to the expansion of the melting PP fibres. On the other hand, the expanding and melting PP fibres provide an essential network of micro-cracks that facilitates the water transport through the tested specimen, which contributes to the reduction of the pore pressure.

**Figure 21:**
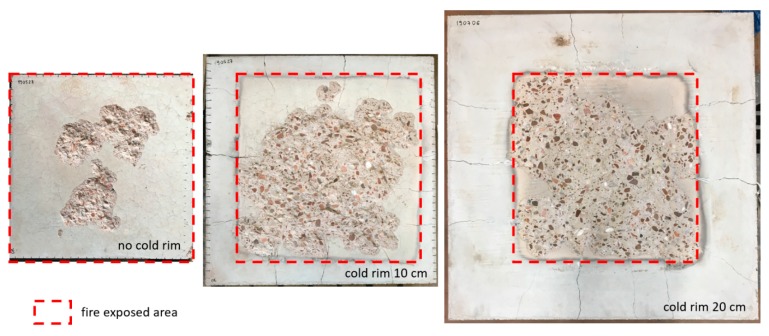
Fire spalling test on HSC concrete slabs with different cold rim widths.

The confinement to thermal strains in concrete can also be introduced by the presence of the unheated part of the concrete slab, which does not expand while heating. Unlike the steel ring, the unheated concrete part counteracts the expansion of the volume of the tested element that is exposed to inert fire. The authors have carried out the experimental tests on three different sizes of HSC slabs (0.6 × 0.6 × 0.15 m^3^, 0.8 × 0.8 × 0.15 m^3^, and 1.0 × 1.0 × 0.15 m^3^), which were exposed to fire. In each case, the area exposed to fire was equal to 0.6 m × 0.6 m. For each tested case, the area exposed to fire was surrounded by an unexposed belt of different widths (0 cm—no cold rim, 10 cm and 20 cm), which constituted a cold rim, as shown in Figure 21. The slab-like element is simply supported at four edges and heated from the bottom in an ISO-834 fire scenario. The results of this test show that concrete is more prone to spalling when a restraint in the form of a cold rim is present. This experiment indicated that, when intermediate scale tests are performed on concrete used for slab-like elements, the test should be conducted on slabs with a cold rim.

**Figure 22:**
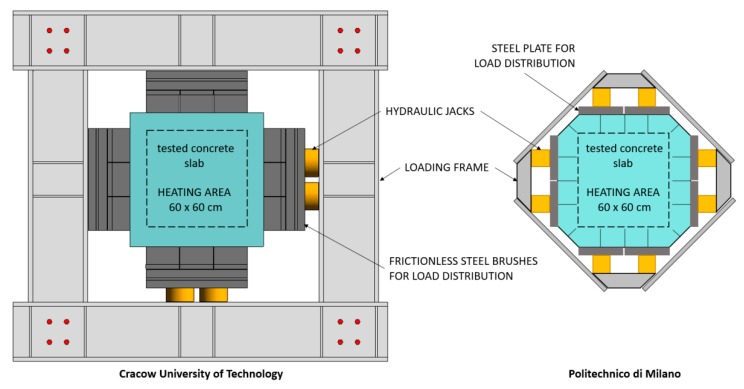
Experimental setup for testing the fire spalling of concrete under an external load action. Left: uniaxial and biaxial loading frame at Cracow University of Technology. Right: biaxial loading frame at Politecnico di Milano [21].

Another type of test that is used to reproduce concrete element behaviour in fire conditions using smaller-scale test procedures consists of testing mechanically-loaded slab-shaped specimens. The external load is introduced in the in-plane lateral direction on a medium-sized slab. The external load reflects the stress state that one can find in real structures (uniaxial or biaxial compression). By choosing the load level, both the restraint and additional external load may be introduced into the testing element. Commonly, the experimental setup consists of a gas furnace, with the opening on the top, on which the slab specimen is placed. Most often, squared slab-like elements are used for this kind of test. An external load is applied to the slab using hydraulic jacks, which are located on the sides of the slab, as shown in Figure 22.

The effectiveness of the PP fibres in fire spalling mitigation of concrete elements may be evaluated because the external biaxial load prevents cracking. Thus, the load controls crack opening and moisture release.

The test, considering the effectiveness of PP fibres using loaded slabs, was presented by Lo Monte et al. in 2015 [21]. The HSC (f_c_ ± 60MPa) slabs, which were 0.8 × 0.8 × 0.1 m^3^ in size, were subjected to constant in-plane lateral compression of 10 MPa during the test. In one tested composition, the addition of 2 kg/m^3^ of PP fibres was used. Slabs were exposed to an ISO-834 fire scenario from the bottom side in an area of 0.6 × 0.6 m^2^. The test results are very similar to those obtained with the above-mentioned ring test. PP fibres do not prevent spalling but strongly reduce its occurrence and help protect the reinforcing steel. Both tests confirm that PP fibres can reduce spalling only to some extent and tests on spalling behaviour and the effective dosage of PP fibres, before designing concrete structures, are strongly recommended.

## 5. Conclusions

This paper presents an overview of current knowledge about the concrete spalling in fire and the role of polypropylene fibres in its effective mitigation. The main mechanisms leading to spalling occurrence include the thermal-mechanical and hygro-thermal mechanisms. The risk of concrete spalling arises together with an increase in concrete strength and density and is practically absent in low-grade concretes characterised by high degrees of open porosity. New-generation high-performance and ultra-high-performance concretes are particularly susceptible to the risk of fire spalling. The use of polypropylene fibres is recommended in order to limit spalling occurrence.

Despite the numerous experiments conducted, the complex numerical hygro-thermal models developed. It is still not possible to effectively determine the risk of fire spalling for concrete with a given composition and properties. Spalling is a complex process that is affected by the composition of concrete (w/c ratio, type of aggregate) and its properties (gas permeability, density, and open pores network) and the spalling risk must be evaluated for a given application.

The effectiveness of polypropylene fibres depends on their melting temperature, geometry length, and cross-section. We have shown that melting processes of fibres cause the creation of micro-cracks and contributes to the lower water vapour pressure. The melting temperature of PP fibres (170 °C) is very close to the peak measured by a number of experimental peaks of pore pressure created in a concrete section exposed to fire. Therefore, the addition of PP fibres to concrete provide successful results in reducing spalling risk due to fire.

The quantity of fibres enabling the percolation of open pores in the matrix, which limits the gas pore pressure picks and prevents spalling, must be determined, and the experimental techniques are commonly used for this purpose. In addition, the manner in which concrete is loaded and the stress conditions during fire (presence of compressive stresses and restraint thermal expansion) plays an important role that needs to be considered when spalling risk is evaluated. Several examples of different approaches for assessing spalling risk in concrete due to fire were presented. It is undeniable that determining mechanical and physical properties of concrete under elevated temperature are vital in the design process. However, in the case of structures being at high risk of fire occurrence, it is recommended to provide the assessment of concrete susceptibility to spall due to fire exposure. The most appropriate approach for the fire test is to ensure the conditions that reflect the intended use of a targeted concrete element.

The complex nature of the problem discussed, the absence of standard procedures for testing the susceptibility of concrete to fire spalling, and the stochastic nature of the process will undoubtedly result in further work on spalling and methods of spalling risk mitigation.

## Figures and Tables

**Table 1:** Spalling test results for plain RPC and RPC modified with PP fibres.

RPC	Heating Rate				
	0.5 °C/min	1.0 °C/min	2.0 °C/min	4.0 °C/min	8.0 °C/min
	Spalling: Yes/No, [Tested Spec./Spec. That Spalled], Spalling Temperature T_spall_				
RPC	no [10/0]	yes [4/4] (241, 261, 254, 241 °C)	yes [4/4] (253, 262, 253, 261 °C)	-	-
RPC PP 1 kg/m^3^	no [10/0]	no [2/0]	no [2/0]	yes [2/1] (331 °C)	yes [2/2] (354, 348 °C)
RPC PP 2 kg/m^3^	no [10/0]	no [2/0]	no [2/0]	no [2/0]	no [2/0]
